# Perioperative Endocrine Therapy for Patients with Cushing's Syndrome Undergoing Retroperitoneal Laparoscopic Adrenalectomy

**DOI:** 10.1155/2012/983965

**Published:** 2012-10-30

**Authors:** Xiaobo Cui, Lu Yang, Jianwei Li, Siyuan Bu, Qiang Wei, Zhenmei An, Tianyong Fan

**Affiliations:** ^1^Department of Urology, West China Hospital, Sichuan University, Chengdu 610041, China; ^2^Department of Urology, Ningbo Medical Treatment Center, Lihuili Hospital, Ningbo 315012, China; ^3^Department of Endocrinology, West China Hospital, Sichuan University, Chengdu 610041, China

## Abstract

*Objectives*. To investigate the efficacy and safety of perioperative endocrine therapy (PET) for patients with Cushing's syndrome (CS) undergoing retroperitoneal laparoscopic adrenalectomy (RLA). *Methods*. The novel, simplified PET modality of 82 patients who underwent RLA procedures for CS were studied. Clinical manifestations were observed for all patients on days 1 and 5 postoperatively, and clinical data, such as blood pressure (BP), levels of serum cortisol, adrenocorticotropin (ACTH), blood glucose, and electrolytes, were acquired and analyzed. *Results*. Supraphysiological doses of glucocorticoid were administered during the perioperative period, and the dosage was reduced gradually. In all 82 cases, the RLAs were performed successfully without any perioperative complication, such as steroid withdrawal symptoms. The patient's symptoms and signs were improved quickly and safely during the hospital days. The serum cortisol and potassium levels were rather stable on days 1 and 5 postoperatively, and most were within the normal range. The clinical manifestations, serum levels of cortisol, ACTH, and potassium in most patients restored to normal gradually after several months (mean, 6.7 ± 1.2 months), except for one patient undergoing bilateral adrenalectomy. *Conclusions*. This perioperative endocrine therapy for patients with Cushing's syndrome (mainly for adrenocortical adenoma) undergoing retro-laparoscopic adrenalectomy is both effective and safe.

## 1. Introduction

Cushing's syndrome (CS) is caused by either hypersecretion of adrenocorticotropin (ACTH, ACTH-dependent Cushing's syndrome) or primary adrenal hypersecretion of glucocorticoids (ACTH-independent Cushing's syndrome). Approximate 85% of CS are ACTH dependent (pituitary adenoma or ectopic adrenocorticotropic hormone syndrome), and about 15% are ACTH independent due to the presence of adrenal lesions (adenoma and carcinoma) or, rarely, to primary bilateral adrenal cortical hyperplasia. The clinical features of CS include centripetal fat distribution, proximal myopathy, purple striae, osteoporosis, hypertension, hypokalemia, and decreased linear growth with continued weight gain in a child. CS could cause significant physical and functional disability and sometimes be fatal if untreated or improperly addressed [1, 2].

When hypercortisolism is caused by adrenal lesions, adrenalectomy is the treatment of choice. Meanwhile, adrenalectomy could not only be the first-line treatment in patients with ACTH-dependent CS but can also palliate symptoms effectively when patients with ACTH-dependent CS have failed other therapeutics. Though operation should be effective in CS treatment, these fragile patients may also endure some potential risks perioperatively [3, 4]. Specifically, patients with overt or subclinical CS undergoing bilateral or unilateral adrenalectomy might confront with some dangers, such as acute adrenocortical insufficiency (AAI), after surgery owning to the long period of hypercortisolemia. Because AAI and some other complications after operation could be life threatening to patients with CS, steroid replacement therapy after adrenalectomy should be optimized to minimize the possible risks. However, replacement therapeutic methods of perioperative hormone maintenance remain controversial up to date [5, 6]. How to establish the most appropriate therapeutic modality to avoid the risks caused by the sharp reduction of cortisol is still of great significance.

Therefore, it would be necessary and prudent to analyze the method, efficacy, and safety of perioperative endocrine therapy (PET) for patients with CS undergoing retroperitoneal laparoscopic adrenalectomy (RLA).

## 2. Materials and Methods

Clinical, laboratory, and pathological data of all 82 patients with CS who underwent RLA were retrospectively analyzed in our center from January 2007 to December 2010. The blood samples were collected and electrochemiluminescence immunoassay (ECLIA) was applied to evaluate the serum cortisol levels perioperatively. The patients' characteristics are summarized and listed in Table 1. All the patients experienced typical features of CS, such as centripetal fat distribution, proximal myopathy, purple striae, and hypertension. All the information, such as symptoms, signs, laboratory data and images of primary adrenal mass on CT or MRI, initially established the diagnosis of ACTH-independent Cushing's syndrome due to adrenal lesions.

All patients underwent RLAs. Particularly, unilateral adrenalectomy was performed in 81 patients (98.8% of all patients), while one patient underwent bilateral RLAs for the bilateral multiple adrenal masses. General anesthesia methods and a lateral decubitus position were applied in all patients during operations. Additionally, any fluctuation of blood pressure (BP) was intraoperatively monitored. Retrolaparoscopic operations were performed with a standardized technique using three trocars at the waist. A carbon dioxide pneumoperitoneum was established with an intra-abdominal pressure of 12–15 mmHg throughout the operation.

Two doses of intravenous 100 mg hydrocortisone were individually given at half an hour prior to the operation and immediately after the removal of the adrenal mass. Then, a dose of 200 mg hydrocortisone was administered in the first 24 h postoperatively. Thereafter, the usages of 100 mg Q8 h, 100 mg Q12 h, and 50 mg Q12 h hydrocortisone were applied, respectively, in the following three days. On the fifth day postoperatively, an oral dose of 25 mg prednisone was subsequently used instead of intravenous agents, followed by a reduction of 5 mg every 3 days until a maintenance dosage (from 10 to 15 mg depending on the patients) was reached. Serum cortisol and ACTH levels, blood glucose levels, electrolytes levels, and BP were each determined, and clinical signs were examined for all patients on days 1 and 5 postoperatively. Further clinical data were collected in the following several months (until the patients' complete recovery).

## 3. Results

When the patients were admitted to hospital, their serum cortisol levels were significantly high ranging from 674.0 to 870.6 nmol/L (mean, 773.4 ± 46.7 nmol/L), and the physiological fluctuations of cortisol were absent. Consequently, the serum ACTH levels were much less than the normal range (0.3 ± 0.1 ng/L; normal, 5.0–7.8 ng/L), and the levels of 24 hr urinary cortisol were markedly high ranging from 497.6 to 1092.5 *μ*g/24 h (mean, 801.2 ± 136.3 *μ*g/24 h). The serum potassium of most patients (68 cases) was lower than normal, ranging from 2.80 to 3.45 mmol/L (mean, 3.22 ± 1.9 mmol/L). 58 patients had fasting blood glucose greater than 6.9 mmol/L before operation, ranging from 6.9 to 13.5 mmol/L (mean, 9.3 ± 2.1 mmol/L) (Table 1).

Retroperitoneal laparoscopic adrenalectomies were successfully performed on all the patients without conversions into open or any perioperative complication, such as AAI. Although all patients had different high levels of BP ranging from 140/90 mmHg to 210/130 mmHg (mean, 156/105 mmHg) before operation and took various types of antihypertensive agents (one, two, or three types of drugs combined) for 2 months to 10 years, the fluctuation of BP did not occur during the surgical procedures. The postoperatively pathological results demonstrated adrenal cortical adenomas in all 82 patients further. Moreover, all the patients were treated with PET as listed above, and the related clinical data were collected.

The postoperative characteristics of the patients involved are summarized in Table 2. On the first day postoperatively, serum cortisol ranged from 61.5 to 182.7 nmol/L (mean, 119.6 ± 25.4 nmol/L) in all patients. Eleven patients (13.4%) took potassium supplements for hypokalemia ranging from 3.07 to 3.46 mmol/L (mean, 3.27 ± 1.3 mmol/L). The serum cortisol levels in these patients with hypokalemia were significantly higher than the patients with normal serum potassium (162.8 ± 12.7 nmol/L versus 110.3 ± 30.1 nmol/L, *P* < 0.05). Meanwhile, eight patients (9.8%) had mild fever (<38°C) and slight skin desquamation. Three patients (3.7%) complained mild nausea and vomiting. No patient complained confusion, coma, severe nausea and vomiting, diarrhea, fatigue, pain, high fever, lethargy, or orthostatic hypotension, which are the common manifestations in serious cortisol withdrawal syndrome.

On the fifth day after surgery, the levels of the patients′ serum cortisol ranged from 133.1 to 302.8 nmol/L (mean, 213.5 ± 47.6 nmol/L), significantly higher than the levels on the first day postoperatively (*P* < 0.05). The cortisol levels in most patients (77, 93.9%) restored to the normal range. The potassium levels were normal in all people (mean, 4.15 ± 3.9 mmol/L). 32 patients (39.0%) needed to take antihypertensive drugs to ensure that BP was kept to less than 140/90 mmHg, with less doses and/or times. Four patients (4.9%) lost weight obviously (5.1–6.8 kg, mean 5.9 ± 0.4 kg). There was not any other special manifestation observed.

Three months after their discharging from the hospital, the patients were all free from CS manifestations. The bluish stretch marks disappeared, all patients lost weight (5.0–13.0 kg, mean, 9.1 ± 2.3 kg), and most patients (75, 91.5%) had normal BP without the assistance of any antihypertensive agent. Four patients (4.9%) took a minimal doze of one antihypertensive drug to ensure the BP normal once a day, and the other 3 patients (3.7%) took the pill once in two days. All patients continued on an oral dose of prednisone from 10 to 15 mg after discharge (37 used 10 mg, 25 used 12.5 mg, and 20 used 15 mg) and were planned to receive steroid replacement therapy for additional several months. Furthermore, this drug therapeutic modality was tapered according to the patients' condition.

Clinical remission (CR) of CS could be defined as the disappearance of clinical symptoms and signs, the serum cortisol, and ACTH levels in the normal range with normal physiological fluctuations, and the normal biochemical data in patients with CS. Patients were followed up to confirm the achievement of CR and no additional oral prednisone required (except for the patient undergoing bilateral adrenalectomy). Most the patients (67 cases) were cured during 6–8 months postpoperatively (12 cases were earlier or 3 cases were later), and the average time was 6.7 ± 1.2 months in this study. The time for cessation of replacement steroids closely followed if everything went well in 3–5 days later. Only 2 persons needed to take one kind of antihypertensive drug to control the BP easily.

## 4. Discussion

Cushing's syndrome can be caused by a variety of diseases with different etiologies and treatment options. However, all of the patients with CS share the common feature of cortisol excess, and the excessive secretion of glucocorticoid can cause a range of metabolic disorders, devastate physical and psychologic conditions, and even increase the risks of cardiovascular events and cerebrovascular accidents [3, 4].

Surgical treatment with adrenalectomy can eliminate the source of the hypercortisolism but may not immediately reverse the consequences of long-term cortisol excess, and acute adrenal insufficiency or steroid withdrawal syndrome (SWS) occurs at times. CS in patients with adrenal lesions inhibits the cortisol secretion through negative feedback in the hypothalamus-pituitary axis (HPA), resulting in decreased ACTH release. Subsequently, the low ACTH could not stimulate and keep the normal state of adrenal cortex around the lesion and the contralateral adrenal gland, usually atrophic in pathology and low in function. Consequently, there would not be adequate cortisol when the adrenalectomy of affected side is completed, and serum cortisol level decreases rapidly [7], sometimes inducing adrenal crisis.

This SWS includes a variety of clinical manifestations, such as disturbance of consciousness, anorexia, nausea, vomiting, diarrhea, weight loss, weakness, myalgia, arthralgia, abdominal pain, headache, lethargy, orthostatic hypotension, fever, and skin desquamation [8, 9]. However, none of these features are specific to SWS. There might be several reasons for the postoperative nausea and vomiting. First, this could be the side effects (SEs) of the analgesia pump. Then, these also might be the common postoperative and post-post-anesthetic manifestations accompanied by no other symptoms, which were easily managed, and no recurrence was observed. Additionally, these symptoms could occur despite that glucocorticoid replacement would be helpful and the serum cortisol levels were stable. This condition could be emergent and fatal when the diagnosis is incorrectly established, and the treatment is improperly given. It is therefore worthwhile to search for the best strategy of perioperative endocrine treatment to prevent the occurrence of the tough SWS perioperatively.

PET is necessary to reduce the physiological and traumatic response during and after the surgical treatment of CS [10]. This perioperative replacement treatment with an ultraphysiological dose of glucocorticoids protects the serum levels of cortisol from the harsh decrease after operation, keeps the normal physiological function, and minimizes the possible risk. Shen et al. [6] followed up 331 patients who underwent surgical resections of the adrenal glands and confirmed that steroid replacement therapy after adrenalectomy should be reserved for the patients with CS (both overt and subclinical). Krikorian et al. [11] insisted that the steroid replacement therapy should be kept during and after surgeries until there is clinical or biochemical evidence of CR.

Although PET during and after adrenalectomy has become a widely accepted therapeutic modality for CS patients, currently there is no agreement on the specific strategy in detail. Different authors may have different opinions on PET selections; however, PET should basically follow the principles of intraoperative and postoperative administration, successive intravenous and oral administration, and then gradual reduction. Orth and Kovaks [12] recommended that 200 mg hydrocortisone could be given during the first 24 h after surgery, and then 100 mg, 75 mg, 50 mg, and a maintenance dose of hydrocortisone (from 10 to 25 mg in the morning) on the following days. Mishra et al. [13] advised that hydrocortisone should be intravenous dripped at the pace of 30 mg/h for 6 h immediately after surgery, and then 200 mg hydrocortisone in total with the pace of 10 mg/h was given on first day postoperatively. During the second day, the treatment modality would be replaced by intravenous infusion of 100 mg hydrocortisone combined with an oral prednisone of 10 mg every 6 h and then took an oral prednisone of 25 mg every 6 h on day 3 postoperatively. The patients were advised to take 20 mg prednisone orally every 6 h on days 4–7 postoperatively, and then gradually the doses were reduced.

The patients were managed to administer two kinds of glucocorticoids in this study. One is hydrocortisone, a drug used intravenously with a half-life of about 1.5 hour, and the other is prednisone, an oral drug with a half-life of about 1 hour. Except for the patients who underwent bilateral adrenalectomy requiring lifelong steroid supplementation, those people with CS who underwent unilateral adrenalectomy can usually be weaned off of all steroids in 6–12 months [14, 15].

RLA for CS has all the advantages of minimal invasion, such as the reduced blood loss, the fewer complications, the more rapid recovery, and the shorter length of hospital stay [16, 17]. Furthermore, the half-life of serum cortisol is rather short, about 80–120 minutes, and 70% of intravenous infused cortisol, or the patient's own cortisol secretion will be cleared within 24 hours. Therefore, it might not be reasonable to start the use of glucocorticoids one day before the surgery on the patients with CS. With our approach to treatment, one dose of intravenous 100 mg hydrocortisone was initially used 30 minutes before surgery, which would help the patients to reach a peak serum level of cortisol when the tumor is being removed and be physiologically ready for operation-related trauma. And then another dose of 100 mg hydrocortisone was given when the neoplasm was resected, which could preserve the normal level of serum cortisol and avoid the occurrence of AAI. The doses and times of intravenous hydrocortisone were gradually decreased, and oral prednisone drug was initiated subsequently. Frankly, although the doses of glucocorticoids reduced (exogenous cortisol), the serum cortisol levels increased steadily on the postoperative day 5. This could be contributed to the more endogenous cortisol produced. Instability of endocrine state and lag effect of exogenous glucocorticoids could be additional reasons. The perioperatively hormonal levels assayed could be insightful, providing the direct figures on the hormonal variations and reflecting the therapeutic effect. Moreover, abnormality of these data could alarm the possibility of SWS. According to the consequences, the serum cortisol levels of our patients remained stable, and the serum ACTH levels increased steadily on days 1 and 5 after surgery. SWS or AAI did not occur during hospital days. Symptoms and signs of these CS patients improved gradually after the RLA for adrenal tumors. All the information above indicate that the simplified PET we introduced is effective on SWS and AAI prevention, safe in use, and easy for implementation.

There are several important points to the present study needed to be clarified. First and foremost, although ECLIA is more accurate, cross-reactivity still existed to some extent owing to the similar molecular structure among various glucocorticoids. Therefore, the serum cortisol levels tested in this study could also stand for some exogenous glucocorticoids.

Then, this retrospective analysis of a clinical protocol on 82 patients could not directly compare with the other methods like RCT. However, this study involving a large number of CS patients still could be meaningful and exert some influence on protocol selection of perioperative steroid treatment. First, no glucocorticoids were applied one day prior to surgeries, no oral drug was used coincided with the intravenous agent, and both the oral and intravenous agents were reduced gradually, which did not resemble the strategies reported by the other experts [12, 13]. Therefore, it is easier for the patients to accept, and practicable for the doctors to master. Second, it would be much better to initiate the oral pills after the complete restoration of the gastrointestinal tract. Thus, the fifth day would be preferred. Third, glucocorticoids drugs with long half-life and potential SEs, such as dexamethasone, were not used in this clinical trial. It would be easy to regulate the usage of the drugs according to the results of the serum cortisol and ACTH levels. Fourth, the strategy was to use less-steroid replacement and reduce the doses gradually, based on the serum hormonal levels. This could help the ACTH secretion by the pituitary gland and the functional and morphologic restoration of the residual adrenal tissue and the contralateral adrenal gland. Additionally, this clinical protocol has been utilized in our department for almost ten years (2003–2012) on over 200 patients with CS. None of them (0/218; 0%) had experienced the AAI, or SWS comparing with 11 of 124 patients (8.9%) in the previous 10 years (1993–2002), which could be a direct proof for the effectiveness and safety of this method.

Last but not least, although there was a trend for less replacement, it seemed that this report did not reflect this directly. Indeed, this study supported this tendency greatly. First, the total doses of glucocorticoids were used less than the other past papers [12, 13]. Second, the whole process of the replacement therapy was simplified, and unnecessary or unreasonable glucocorticoids were not insisted. Additionally, the time to patients' recovery was often reported to be 6–12 months postpoperatively, and oral prednisone should always be persisted fully during that time in many previous studies. In this trial, some patients might have been CR of CS before 6 months postpoperatively, and unnecessary glucocorticoids were no longer asked to take. This could reduce the use of glucocorticoids for replacement.

## 5. Conclusions

The application of the simplified perioperative endocrine therapy for patients with Cushing's syndrome undergoing retroperitoneal laparoscopic adrenalectomy is both effective and safe and provides the surgeons with an affirmative option.

## Figures and Tables

**Table 1:** Patients' characteristics before surgeries.

Items	Patients' information
Number	82 patients
Age	29 ± 4.7 years (range, 18–53)
Gender (*n*[%])	20 male (24.4%); 62 female (75.6%)
Diagnosis by CT or MRI (*n*[%])	
Single adrenal adenoma	80 (97.6%)
Multiple adrenal adenoma (unilateral)	1 (1.2%)
Bilateral adrenal adenoma	1 (1.2%)
Time to diagnosis	18 ± 7.5 months (range, 1–61 months)
Blood pressure when admitted to hospital	156/105 mmHg (range, 140~210/90~130 mmHg)
Hypokalemia (*n* value)	68; 3.22 ± 1.9 mmoL/L (range, 2.80–3.45 mmoL/L)
Fasting blood glucose greater than 6.9 mmoL/L	58; 9.3 ± 2.1 mmoL/L (range, 6.9–13.5 mmoL/L)
physiological fluctuationofcortisol	Absent in all patients
Serum cortisol on 8 AM	773.4 ± 46.7 nmoL/L (range, 674.0–870.6 nmoL/L)
	(normal, 147.3–609.3 nmoL/L)
24 hr urinary cortisol	801.2 ± 136.3 *μ*g/24 h (range, 497.6–1092.5 *μ*g/24 h)
	(normal, 20.26–127.55 *μ*g/24 h)
Serum ACTH 8 AM	0.3 ± 0.1 ng/L, (range, 0.05–0.8 ng/L)
	(normal, 5.0–7.81 ng/L)
High dose dexamethasone suppression test (HDDST)	No suppression in all 82 patients
Low dose dexamethasone suppression test (LDDST)	No case of complete suppression (serum cortisol levels < 50 nmoL/L)
	No suppression in 82 patients (>140 nmoL/L)

**Table 2:** Patients' postoperative characteristics.

Items	Information on day 1 postoperatively	Information on day 5 postoperatively
Serum cortisol on 8 AM (range, mean)	61.5–182.7 nmoL/L	133.1–302.8 nmoL/L
	(119.6 ± 25.4 nmoL/L)	(213.5 ± 47.6 nmoL/L)
Serum ACTH on 8 AM (range, mean)	0.1–1.3 ng/L	0.3–3.0 ng/L
	(0.7 ± 0.3 ng/L)	(1.3 ± 0.5 ng/L)
Hypokalemia (*n*, range, mean)	11, 3.07–3.46 mmoL/L	None
	(mean, 3.27 ± 1.3 mmoL/L)	
Confusionorcoma (*n*)	None	None
Diarrhea (*n*)	None	None
Nausea and vomiting (*n*)	3	None
Headache (*n*)	None	None
Weight loss (*n*)	None	4
Generalized body aching (myalgia, arthralgia, and abdominal pain, *n*)	None	None
Weakness and drowsiness (*n*)	None	None
Orthostatic hypotension (*n*)	None	None
Fever and skin desquamation (*n*)	8 (slight)	None
